# Predictors of intention to use modern contraceptives among female senior secondary school students in the Kpando Municipality, Ghana

**DOI:** 10.4314/ahs.v21i3.49

**Published:** 2021-09

**Authors:** Amanda D Der, Judith A Anaman-Torgbor, Veronica O Charles-Unadike, Elvis E Tarkang

**Affiliations:** 1 School of Public Health, University of Health and Allied Sciences PMB 31 Ho, Ghana; 2 School of Nursing and Midwifery, University of Health and Allied Sciences PMB 31 Ho, Ghana; 3 HIV/AIDS Prevention Research Network Cameroon PO Box 36 Kumba, Cameroon

**Keywords:** Modern contraceptive use, theory of planned behaviour, senior secondary school students, Ghana, Kpando Municipality

## Abstract

**Background:**

Improving the reproductive health of young women in developing countries requires access to safe and effective methods of fertility control. Volta Region records one of the highest prevalence of teenage pregnancy and adolescents aged 15–19 years are the least acceptors of contraceptives in the Region. Guided by the Theory of Planned Behaviour, this study determined predictors of intention to use modern contraceptives among female Senior Secondary School students in the Kpando Municipality, Ghana.

**Method:**

A cross-sectional design was adopted, collecting data among a multistage sample of 270 participants, using a pretested self-administered questionnaire and analysing them using Stata software Version 16 at the 0.05 level of significance.

**Results:**

The mean age of the respondents was 16.78 ± 1.31. About 40.2% of the sexually experienced participants used a modern contraceptive during ther first sexual encounter. However, the majority (69.3%) had the intention to use modern contraceptives. Regarding subjective norms, the majority of the significant others (63.0%) were not supportive of modern contraceptive use and more than half of the respondents (59.3%) had a positive attitude towards modern contraceptive use, while 54.1% perceived that they had control over modern contraceptive use. The majority (69.3%) had the intention to use modern contraceptives. Perceived behavioural control was the only significant predictor of the intention to use modern contraceptives [AOR= 9.80 (C.I: 5.11, 18.77); p< 0.001].

**Conclusion:**

Interventions to increase the perception of control over contraceptive use is of the essence. This will help increase their intention to use modern contraceptives.

## Introduction

Improving the reproductive health of young women in developing countries requires access to and use of safe and effective methods of fertility control; hence special attention is needed to ensure that the contraceptive needs of vulnerable groups such as unmarried young women, adolescents, poor women and rural women are met to reduces inequities in access^1^,^2^.

The World Health Organization defines adolescents as individuals in the 10–19 years' age group and youth as the 15–24-year age group. Adolescents and youth form a majority of sexually active individuals in a population. Many of these young people could be at risk of the consequences of unprotected sexual intercourse and sexually transmitted infections (STIs), especially HIV/AIDS. Adolescent females aged 15–19 account for over 14 million births each year, 91% of these are in low and middle-income countries^3^. The lack of contraceptive use among adolescents can lead to an increase in several unwanted pregnancies, abortions and STIs^4^. Modern contraceptive use has the potential of improving the quality of life of adolescents and their economic welfare^5^. Between 2012 and 2017, the number of women of reproductive age who were married or in a union who use modern contraceptive methods increased by 28.8 million globally. At the regional level, modern contraceptive prevalence among women of reproductive age in Asia who were married or in union grew from 51.0% to 51.8% between 2012 and 2017, which is a slow growth, particularly when compared with a change from 23.9% to 28.5% across Africa^6^.

According to the Theory of Planned Behaviour (TPB), behaviours can be predicted with high accuracy from positive attitudes toward the behaviour, supportive subjective norms, and high perceived behavioural control; and these with strong perceived behavioural intentions account for considerable variance in actual behaviour^7^,^8^. The perceived behavioural control accounts for about 65% of the variance for intention and 27% of the variance in behaviour among women^9^. Further, the probability of using modern contraceptives among orthodox Christian women is 4.22 in some parts of Africa^10^.

The proportion of modern contraceptive acceptors increased gradually from 18% in 2009 to 23% in 2014 in the Volta Region of Ghana with the age group 15–19 being the least acceptors^11^. The TPB predicted 26.0% of the variance in the use of contraceptives in a reproductive health study conducted in Uganda^12^. According to a meta-analysis conducted to explore whether the constructs in the TPB explain condom use behaviour, finding revealed that attitude, subjective norm and perceived behavioural control accounted for 24.0% of the variance in condom use intention and were all significant correlates. Intention and perceived behavioural control accounted for 12.4 % of the variance in condom use behaviour^13^.

Evidence has shown that attitudes towards condom use and perceived behavioural control over condom use was significantly positively association with the intention to use it among in-school heterosexual youth in the Eastern region of Ghana^14^. Also, the intention to use condoms predicted condom use behaviour among the youth. Moreover, intention to use condoms mediated the attitude-behaviour relationship, and the perceived control-behaviour relationship. This highlights the importance of using behavioural beliefs, perceived control beliefs and behavioural intention as key variables in condom promotion programmes among in-school heterosexual youth in the Eastern region of Ghana. Further, the intentions to use condoms and past condom use behaviour accounted for a significant proportion of the variance in future condom use behaviour among young Ghanaians^15^. Past condom use also moderated the future condom use intention-behaviour relationship, indicating that it is useful to consider young people's past experiences with condoms in informing the design of condom use skills training^15^.

**Figure 1:**
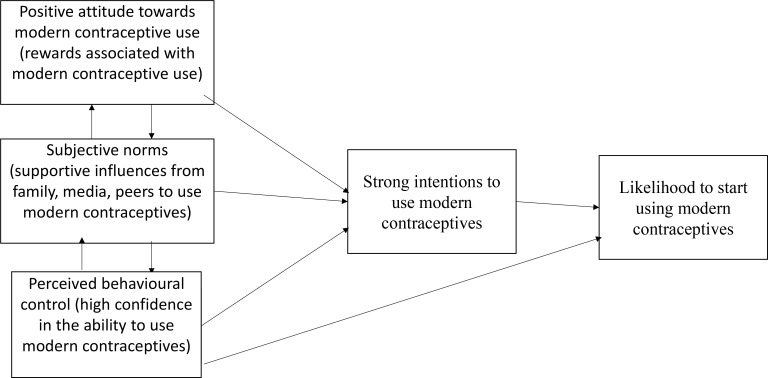
Conceptual framework: Adapted from Ajzen (1991)^7^

It has also been reported that as predicted by the TPB, attitudes were significantly positively associated with intentions to use condoms over time^16^. However, subjective norms were not significantly associated with intentions to use condoms over time. Perceived control did not predict intentions to use condoms over time^16^. Also, attitudes towards condom use were more favourable among male students than they were among female students. Male students perceived slightly greater control over condom use than did female students^17^. A broad understanding of these factors has far-reaching significance for policy makers, researchers, and health care professionals and planners to develop adolescent sexual and reproductive health programmes that address the relevant factors of modern contraceptive use intentions. This could subsequently influence modern contraceptive use among adolescents and youths. Guided by the TPB (Figure 1), this study, therefore, determined the predictors of the intention to use modern contraceptives among female Senior Secondary School (SSS) students in the Kpando Municipality in the Volta region of Ghana. This Municipality falls in the Volta Region, where adolescents aged 15–19 years are the least contraceptive acceptors. This study is necessitated by the paucity of research in the Municipality on modern contraceptive use among adolescents and young adults. Results of this study will inform policies aimed at increasing use of modern contraceptive and in turn reducing adolescent pregnancy complications.

## Hypotheses

The following hypotheses have been derived from the TPB to guide this research:

1. Female students with a positive attitude towards modern contraceptive use will have the intention to use them to prevent unwanted pregnancy and STIs.

2. Female students with a positive subjective norm regarding modern contraceptive use, will have the intention to use them to prevent unwanted pregnancy and STIs.

3. Female students, who perceive control modern contraceptive use, will have the intention to use them to prevent unwanted pregnancy and STIs.

## Methods

### Study site

According to the Ghana Statistical Service18, out of the total population of 53,736 in the Kpando Municipality, people between the ages of 15 and 19 years make up 5,827, representing 10.8% with 2,917 males representing 11.3% and 2,910 females representing 10.5%. About 90.3% of the adolescents have never married and the fertility rate of female adolescents in the Municipality is 325. There are two Senior Secondary Schools in the Kpando Municipality and 8.6% of adolescents are currently attending Senior Secondary Schools^18^. There are two hospitals, five health centres, seven Community-based Health Planning and Services (CHPS) compounds, one Reproductive Health Centre and two maternity homes in the Municipality. All the health facilities provide some form of contraception counselling and services to the people.

### Study population

Only female students in SSSs in the Kpando Municipality were involved in this study. This is because they are considered being at a higher risk of unwanted pregnancy, unsafe abortions and STIs.

### Inclusion and exclusion criteria

#### Inclusion criteria

The study included female students who were studying in the Senior Secondary Schools in the Kpando Municipality and were available at the time of the study. Consent was sought from students who were 18 years and above and parental consent and child assent were sought from students who were below 18 years at the time of the study. Teachers, however, gave parental consent to students since they were in a boarding institution.

#### Exclusion criteria

Students were excluded if they were severely ill.

### Study design

A quantitative study approach employing cross-sectional design was used for this study and data was collected in February 2020^19^.

### Sample size determination and sampling method

The sample size for this study was calculated using the single proportion population formula by Cochran^20^, The sample size was estimated based on 21% (0.21) prevalence of modern contraceptive use among women of reproductive age in Ghana21. A confidence interval of 95%, a significance level of 5% (0.05 margin of error), a Z-score of 1.96 and a 5% non-response rate was used.

Hence, sample size,, n = 254.9286

Adjusting for anticipated 5% non-response rate, n = (254.9286 x 0.05) + 254.9286 = 12.7464 + 254.9286 = 267.6750.

A minimum of 268 students were estimated; however, 270 students were recruited for the study using the multistage sampling technique and this involved taking proportionate samples from the two SSSs in Kpando Municipal that had female students. The first stage involved purposively selecting the two schools that had female students. At the second stage, each class was treated as a stratum namely, SSS 1, SSS 2 and SSS 3. To ensure equal representation, proportionate samples were taken from each school and an equal number of female students were then selected from each class. Female students who met the inclusion criteria were then selected from each stratum through simple random sampling. Female SSS 2 students in school B were not present at school during the data collection due to the double-track system and hence the total female population of only SSS 1 and 3 was used. Based on the female populations, 120 female students were recruited in School B while 150 female students were recruited in School A. This was done by writing ‘Yes’ and ‘No’ on pieces of paper which were then squeezed and mixed and students who met the inclusion criteria were allowed to pick. Students who picked the ‘Yes’ were automatically part of the study.

### Data collection procedure

A standardised pretested structured questionnaire was used to collect the data for the current study^8^. The questionnaire was self-administered and contained two sections. Section A contained 6 questions on demographic characteristics of respondents while section B contained 14 questions on factors that influence modern contraceptive use intention based on the constructs of the TPB respectively. Due to the sensitive nature of the topic, two female research assistants of a similar educational level were trained to assist in the data collection so as to avoid any influences such as shyness in answering the questions. The two females had simiar educational background to ensure equal understanding of the questionnaire. Understanding the nature and scope of the questionnaire is expedient in helping participants to understand the questions and answer them. Informed consent was obtained from participants who were 18 years and above and those below 18 years signed a child assent form after their guardians had signed the parental consent form before they were included in the study. The teachers served as the guardians as students were in a boarding house. The questionnaires were pre-tested among 20 female students in a different SSS other than the study population to test the feasibility and reliability of the data collection instrument and all weaknesses of the instrument were improved before the actual data collection. It took participants about 20 minutes to complete the questionnaire.

### Measures

The average score of each section was used as a guide in generating the scores. The composite scores were computed for each of the constructs of TPB. On the construct ‘perceived behavioural intention’, one question was asked: ‘will you consider using modern contraceptives in the nearest future?’ Those who responded ‘yes’ were considered to have the intention of using modern contraceptives and those who responded ‘no’ were considered to have no intention of using modern contraceptives.

Three (3) items were used to measure the construct ‘subjective norm’. The response options ‘No’ and ‘Don't know’ were combined to be ‘No’; also, the ‘can't remember’ responses were added to the ‘No’ responses for the question: “did your parents ever asked you to use modern contraceptives”. This was to generate a composite score for the subjective norm. Respondents who had below the average score were considered as having a non-supportive subjective norm and those who scored the average and above were considered as having a supportive subjective norm.

Five (5) items were used to assess participants' attitude towards contraceptive use behaviour and the average score was four (4). All the ‘No’ and ‘Don't know’ responses for all questions were combined to be ‘No’ to generate a more composite score out of the 5 total scores. Those who scored below 4 were considered to have a negative attitude towards contraceptive use behaviour and those who scored 4 and above were said to have a positive attitude towards contraceptive use behaviour.

On the construct ‘perceived behavioural control’, five (5) questions were employed. The average score was computed to be 3 and therefore, respondents who scored below 3 were considered to have a weak perceived behavioural control and those who scored 3 and above were considered to have a strong perceived behavioural control.

### Data analysis

Data were analysed using Stata software version 16. Descriptive statistics such as frequencies and percentages were used to summarize the data. Binomial logistic regression analysis was performed to determine the predictors of intention to use modern contraceptives based on the TPB at the 0.05 level of significance and 95% confidence interval.

All the statistically significant independent variables (the constructs of the TPB) at bivariate analyses (Chi-square) were subsequently added to the logistic regression models one at a time while adjusting for the confounding effects of other independent variables (the constructs of the TPB).

### Ethical issues

Ethical approval was obtained from the University of Health and Allied Sciences Research Ethics Committee (UHAS-REC A.4[10] 19–20. Permission to conduct this study was also obtained from the Kpando Municipal Education Service and the administration of each school. Participation was voluntary and students could withdraw from the study at any time they wished. Names and details of participants were not linked to the data analysis and the findings to ensure anonymity. Confidentiality was ensured by not disclosing information about participants to anybody.

## Results

### Socio-demographic characteristics of the respondents

A total of 270 female students in SSSs in the Kpando Municipality took part in the study and all the questionnaires were returned giving a response rate of 100%. Out of this, 150 (55.6%) were from school A. The majority of the respondents 192 (71.1%) were between the ages of 12 and 17 years. Christians also represented the majority of the respondents 251 (93.0%). Among the respondents, 213 (78.9%) were not in any intimate relationship and the majority belonged to the Ewe ethnic group 215 (79.6%). Most of the respondents were in SSS one (1) and SSS three (3) both representing 40.7% each while SSS two (2) had 50 (18.6%) with 124 (46.0%) studying home economics as shown in Table 1.

**Table 1:** Socio-demographic characteristics of the respondents (n=270)

Variable	Frequency (percentage) N (%)
**School**	
School A	150 (55.6)
School B	120 (44.4)
**Age (Years) Mean age (16.78 ± 1.31)**	
12–17	192 (71.1)
18–22	78 (28.9)
**Religion**	
Christianity	251 (93.0)
Islam	19 (7.0)
**In intimate relationship**	
No	213 (78.9)
Yes	57 (21.1)
**Ethnic group**	
Ewe	215 (79.6)
Others	55 (20.4)
**Class (Grade level)**	
SSS 1	110 (40.7)
SSS 2	50 (18.6)
SSS 3	110 (40.7)
**Program of study**	
General art	77 (28.5)
Technical skills	43 (15.9)
Home economics	124 (46.0)
Others	26 (9.6)

SSS: Senior Secondary School

### Predictors of the intention to use modern contraceptives based on the Theory of Planned Behaviour

The predictors of the intention to use modern contraceptives based on the constructs of the theory of planned behaviour are shown in Table 2. Overall, the subjective norms of respondents were not supportive towards modern contraceptive use behaviour 170 (63.0%), while the attitude towards modern contraceptive use was positive 160 (59.3%) and the perceived behavioural control for modern contraceptive use was strong 146 (54.1%).

**Table 2:** Predictors of the intention to use modern contraceptives based on the Theory of Planned Behaviour (n=270)

Variable	Frequency (Percentage) N (%)
**SUBJECTIVE NORMS**	
**Parents ever asked you to use modern contraceptives**	
Yes	33 (12.2)
No	222 (82.2)
Can't remember	15 (5.6)
**Influenced by your friends who use modern contraceptives to also use**	
Yes	58 (21.5)
No	193 (71.5)
Don't know	19 (7.0)
**Social media, television, radio, newspapers affect the type of modern contraceptives you use**	
Yes	48 (17.8)
No	141 (52.2)
Don't know	81 (30.0)

**SUBJECTIVE NORMS SCORE**	
Not supportive	170(63.0)
Supportive	100(37.0)

**ATTITUDE TOWARDS MODERN CONTRACEPTIVE USE**	
**Modern contraceptive use can effectively prevent teenage pregnancy**	
Yes	213 (78.9)
No	25 (9.3)
Don't know	32 (11.9)
**Modern contraceptive use can prevent abortion and its complications**	
Yes	171 (63.3)
No	40 (14.8)
Don't know	59 (21.9)
**Some modern contraceptives can prevent both pregnancy and STIs**	
Yes	192 (71.1)
No	29 (10.7)
Don't know	49 (18.2)
**Drop out of school if you get pregnant**	
Yes	255 (94.4)
No	15 (5.6)
**Parents will disown you if you get pregnant and still in school**	
Yes	156 (57.8)
No	58 (21.5)
Don't know	56 (20.7)

**ATTITUDE TOWARDS CONTRACEPTIVE USE SCORE**	
Negative attitudes	110(40.7)
Positive attitudes	160(59.3)

**PERCEIVED BEHAVIOURAL CONTROL**	
**Can convince your partner to use modern contraceptives**	
Yes	160 (59.3)
No	110 (40.7)
**Confident to go to a health facility for modern contraceptives**	
Yes	130 (48.2)
No	140 (51.9)
**Confident that modern contraceptives will prevent unwanted pregnancy and STIs**	
Yes	147 (54.4)
No	123 (45.6)
**Confident in using modern contraceptives**	
Yes	107 (39.6)
No	163 (60.4)
**Refuse partner sex if they do not agree for you to use modern contraceptives**	
Yes	181 (67.0)
No	89 (33.0)

**PERCEIVED BEHAVIOURAL CONTROL SCORE**	
Weak perceived behavioural control	124 (45.9)
Strong perceived behavioural control	146 (54.1)

Of the respondents, 82 (30.4%) reported to have had sex before, of which 33 (40.2%) used a modern contraceptive during their first sexual intercourse. However, most of the respondents, 187(69.3%) had the intention of using modern contraceptives.

### Logistic regressions of the predictors of intention to use modern contraceptives based on the Theory of Planned Behaviour

Logistic regression analysis of predictors of the intention to use modern contraceptive based on the constructs of the TPB is presented in Table 3. On subjective norm, respondents whose parents had never asked them to use a modern contraceptive method were 94% less likely to have the intention to use it [AOR=0.06 (C.I: 0.01, 0.44); p= 0.006]. However, the overall subjective norm was not a significant predictor of the intention to use modern contraceptives.

**Table 3:** Logistic regressions of predictors of the intention to use modern contraceptives based on the Theory of Planned Behaviour

Variable	Behavioural intention		Chi-square () (p-value)	COR (95% C.I) (p-value)	AOR (95% C.I) (p-value)

	No intention n(%)	Intention n(%)			
**Subjective Norms**					
Parents asked you to use modern contraceptives					
Yes	1(1.2)	32(17.1)		Ref	Ref
No	80(96.4)	142(75.9)		0.55(0.01, 0.41)0.005	0.06(0.01, 0.44)0.006
Can't remember	2(2.4)	13(6.9)	16.96(0.000)	0.20(0.02, 2.44)0.209	0.20(0.02, 2.48)0.212
**Subjective Norm Score**					
Not supportive	63(75.9)	107(57.2)		Ref	Ref
Supportive	20(24.1)	80(42.8)	8.61(0.003)	2.36(1.32, 4.21)0.004	1.50(0.78, 2.90)0.226
**Attitude Towards** **Contraceptive** **Behaviour Score**					
Negative	33(39.8)	77(41.2)			
Positive	50(60.2)	110(58.8)	0.05(0.827)		
**Perceived Behavioural** **Control**					
Convince your partner					
Yes	28(33.7)	132(70.6)		Ref	Ref
No	55(66.3)	55(29.4)	32.34(0.000)	0.21(0.12, 0.37)0.000	0.20(0.11, 0.36)<0.001*
Confident to go to a health facility					
Yes	12(14.5)	118(63.1)		Ref	Ref
No	71(85.5)	69(36.9)	54.48(0.000)	0.10(0.05, 0.20)0.000	0.08(0.04, 0.17)<0.001*
Confident that modern contraceptives will prevent unwanted pregnancy and STIs					
Yes	28(33.7)	119(63.6)		Ref	Ref
No	55(66.3)	68(36.4)	20.72(0.000)	0.29(0.17, 0.50)0.000	0.26(0.15, 0.45)<0.001*
Confident in using modern contraceptives					
Yes	10(12.1)	97(51.9)		Ref	Ref
No	73(88.0)	90(48.1)	38.11(0.000)	0.13(0.06, 0.26)0.000	0.11(0.05, 0.23)<0.001*
Refuse partner sex					
Yes	38(45.8)	143(76.5)		Ref	Ref
No	45(54.2)	44(23.5)	24.50(0.000)	0.26(0.15, 0.45)0.000	0.25(0.14, 0.44)<0.001*
**Perceived Behavioural** **Control Score**					
Weak	68(81.9)	56(30.0)		Ref	Ref
Strong	15(18.1)	131(70.0)	62.55(0.000)	10.60(5.59, 20.13)0.000	9.80(5.11, 18.77)<0.001*

COR (Crude Odds Ratio), AOR (Adjusted Odds Ratio), C.I (Confidence Interval)

* (Predictors of modern contraceptive use)

On the construct perceived behavioural control, respondents who were not able to convince their partners to use a modern contraceptive were 80% less likely to have the intention to it [AOR=0.20 (C.I: 0.11, 0.36); p< 0.001]. Also, those who were not confident to go to a health facility for a modern contraceptive were 92% less likely to have the intention to use it [AOR= 0.08 (C. I: 0.04, 0.17); p< 0.001] and respondents who had no confidence that modern contraceptives will prevent unwanted pregnancy and STIs were 74% less likely to have the intention of using them [AOR= 0.26 (C.I: 0.15, 0.45); p< 0.001]. Respondents who were not confident that they can use a modern contraceptive were 89% less likely to have the intention to use it [AOR= 0.11 (C.I: 0.05, 0.23); p< 0.001] and respondents who said they would not be able to refuse their partners sex if they did not agree for them to use a modern contraceptive method were 75% less likely to have the intention to use [AOR= 0.25 (C.I:0.14, 0.44); p< 0.001]. Overall, respondents with a strong perceived behavioural control over modern contraceptive use were 10 times more likely to have the intention to use them [AOR= 9.80 (C.I: 5.11, 18.77); p< 0.001].

## Discussion

The utilisation of modern contraceptives is effective in the reduction of unwanted pregnancies and STIs. The intention to use modern contraceptives is especially important among female SSS students as it will greatly influence their contraceptive use behaviour. The current study employed the TPB to determine the predictors of respondents' intention to use modern contraceptives.

From the current study, the proportion of respondents with the intention to use modern contraceptives was 69.3%. This is consistent with a study conducted in Uganda and other studies conducted in Ghana which reported the intention to use modern contraceptives of 57%, 88.34% and 56% respectively^12^, ^22^, ^23^. This suggests that the TPB may be a suitable theory to understand the use of modern contraceptives as the intention is the closest antecedent to behaviour. This, when translated to behaviour would lead to the prevention of unwanted pregnancies, unsafe abortions and STIs, especially HIV/AIDS among adolescents.

This study revealed that subjective norms, thus the support from parents, significant others and the media to take up contraceptive use behaviour was low (37.0%). This is in contrast with a study conducted in Uganda, which reported that subjective norms were fairly supportive of contraceptive use (60%)^12^. The current finding could be because most respondents were adolescents and hence their parents would not ascribe to them being in sexual relationships. It could also be that the cultural setting of the current study does not encourage issues bothering on sexuality to be discussed between parents and their wards. As revealed in the current study, the majority of respondents reported that their parents had never asked them to use modern contraceptives. It is also possible that though they might be using modern contraceptives, their friends would not be aware and have any influence on their choices as they would want to have a sense of belonging especially when those friends are not sexually active. The low supportive subjective norms could also render them to make the wrong choices about modern contraceptives, leading to unwanted pregnancies and the contraction of STIs. Campaigns must be made to educate parents on the need to talk about sex and contraceptive use with young people.

There was a positive attitude towards modern contraceptive use (59.3%) though relatively low as compared to the study conducted in Uganda, which reported a positive attitude of 63.5%^12^. Similarly, perceived behavioural control was strong (54.1%) as compared to relatively low perceived behaviour control (41.3%) reported in the Uganda study^12^. The current finding is notable as respondents would desire to use modern contraceptives to postpone pregnancy and be free from STIs to focus on their academic work. From the curent study, it implies that respondents had the perception that they would be able to use modern contraceptives. This, to an extent, could be an accurate reflection of actual behavioural control and can together with intentions predict behaviour.

The output of binomial logistic regression to determine the predictors of the intention of respondents to use modern contraceptives revealed only perceived behavioural control to be a significant predictor. Respondents who said they could not convince their partners to use modern contraceptives were less likely to use them.

This could be attributed to male dominance in sexual decision-making in the Ghanaian setting. Respondents who had no confidence in going to a health facility to obtain moderns contraceptives were also less likely to use them. This could mean that respondents might be shy to get themselves a modern contraceptive method even if they intended to use them. This, however, could ultimately lead to unwanted pregnancies and probably unsafe abortions and its associated complications. In the same vein, respondents who had no confidence that modern contraceptives could prevent unwanted pregnancies were less likely to use them. This perception could be due to entrenched beliefs as all modern contraceptives have an accepted efficacy of preventing unwanted pregnancies. Health education in this regard should be intensified to change this perception of female students. Respondents who had no confidence in using modern contraceptives and those who said they could not refuse their partners sex if they did not agree to use modern contraceptives were less likely to use them. Females need to have the ability to negotiate sex if it would put them at risk of unwanted pregnancies and STIs and that can only be achieved if they are well empowered with information to make decisions that concern their reproductive health. Overall, respondents who had a strong perceived behavioural control over the use of modern contraceptives were more likely to use them. This is consistent with studies conducted in Uganda^12^, Iran^9^ and several studies conducted in Ghana^14^, ^16^, ^23^. This finding implies that it is important to focus on empowerment programmes such as career guidance and counselling programmes and adolescent health clubs in SSSs to increase the convictions of adolescent girls to increase their intention to use modern contraceptives during every sexual encounter to prevent unwanted pregnancies and possible STIs.

However, this study revealed no significant association between subjective norms and the intention to use modern contraceptives. This is contrary to studies conducted in Uganda^12^, in Iran^9^, Northern Ethiopia^10^ and Ghana^16^. This discrepancy could be because respondents in the current study who said their parents had never asked them to use modern contraceptives were less likely to use them. This finding could be because of the inadequate discussions on sexual and contraceptive use between parents and their children as a result of taboos in the setting of the current study. Also, as the study population is largely adolescents, it could be said that they want to feel independent and therefore do not greatly allow friends and social media to influence their choice of contraceptive even though these shaped their behaviours.

The attitude towards contraceptive use was also not significantly associated with the intention to use modern contraceptives. This is in contrast with studies conducted in Uganda^12^, in Iran^9^ and in Ghana^14^, ^17^ and could be attributed to misinformation on the various types of modern contraceptives available, the correct use of each method and the effects of not using a modern contraceptive method. This could be alarming as it may lead to an increase in adolescent pregnancies, unsafe abortions and STIs.

The current findings should be interpreted in light of the following limitations. First, the sensitive nature of the questions in the current study could have the potential of introducing social desirability bias to the responses. Second, the moral beliefs of society concerning sexuality, especially among young women could influence respondents' ability to answer the questions in morally acceptable ways. The study did not include male students, their inclusion might have explained another dimension in decision-making regarding contraceptive use. However, confidentially was assured and participants were encouraged to individually respond to the questions and were made aware that their responses were needed only for the research. These might have minimised the limitations.

## Conclusion

The subjective norms were not supportive of the utilisation of modern contraceptives. However, respondents had fairly positive attitudes toward contraceptives behaviour, strong perceived behavioural control and most of them had the intention to use modern contraceptives. This may present an opportunity for SSSs to be targeted for education on reproductive health. Furthermore, perceived behavioural control was only the significant predictor of the intention to use modern contraceptives. This implies that, even without supportive subjective norms from parents, friends and close relations, respondents who had strong perceived behavioural control were more likely to have the intention to use modern contraceptives regardless of their attitude. Health promotion programmes for students should be made to empower them to help develop their intentions of using modern contraceptives.
